# A pyoverdine-based iron biochelate from bacterial secretions as an effective fertilizer under alkaline conditions

**DOI:** 10.3389/fpls.2025.1675837

**Published:** 2025-12-15

**Authors:** José María Lozano-González, Juan José Lucena, Sandra López-Rayo

**Affiliations:** Department of Agricultural Chemistry and Food Science, Universidad Autónoma de Madrid, Madrid, Spain

**Keywords:** biofertilizer, pyoverdine, Pvd/Fe3+, *Pseudomonas*, biostimulant

## Abstract

**Introduction:**

Iron (Fe) deficiency in crops grown on calcareous soils seriously limits their productivity. The most common practice to address this issue is the application of Fe-chelates. However, more environmentally friendly methods are now being adopted. This study evaluated a pyoverdine-based siderophore extract from *Pseudomonas* RMC4 (Pvd) as an Fe biofertilizer.

**Methods:**

Stability batch tests and plant assays were conducted, including ferric chelate reductase (FCR) activity, ⁵⁷Fe-labeled uptake, phytotoxicity, and a hydroponic test with cucumber plants at two Fe levels (5 and 10 µM).

**Results and discussion:**

The Pvd/Fe³⁺ biofertilizer showed high stability at alkaline pH and resistance to Ca²⁺ interference. Although only a limited reduction of Pvd/Fe³⁺ by FCR was observed, Fe showed efficient translocation to shoots. Pvd/Fe³⁺ improved root morphology and biomass, and decreased reactive oxygen species, demonstrating biostimulant effects. At 10 µM, Pvd/Fe³⁺ provided Fe supply efficiency comparable to commercial HBED/Fe³⁺, and improved Fe/Mn ratio. These findings support the potential of Pvd/Fe³⁺-based formulations as dual-function biofertilizers and biostimulants.

## 1 Introduction

**Figure 1:**
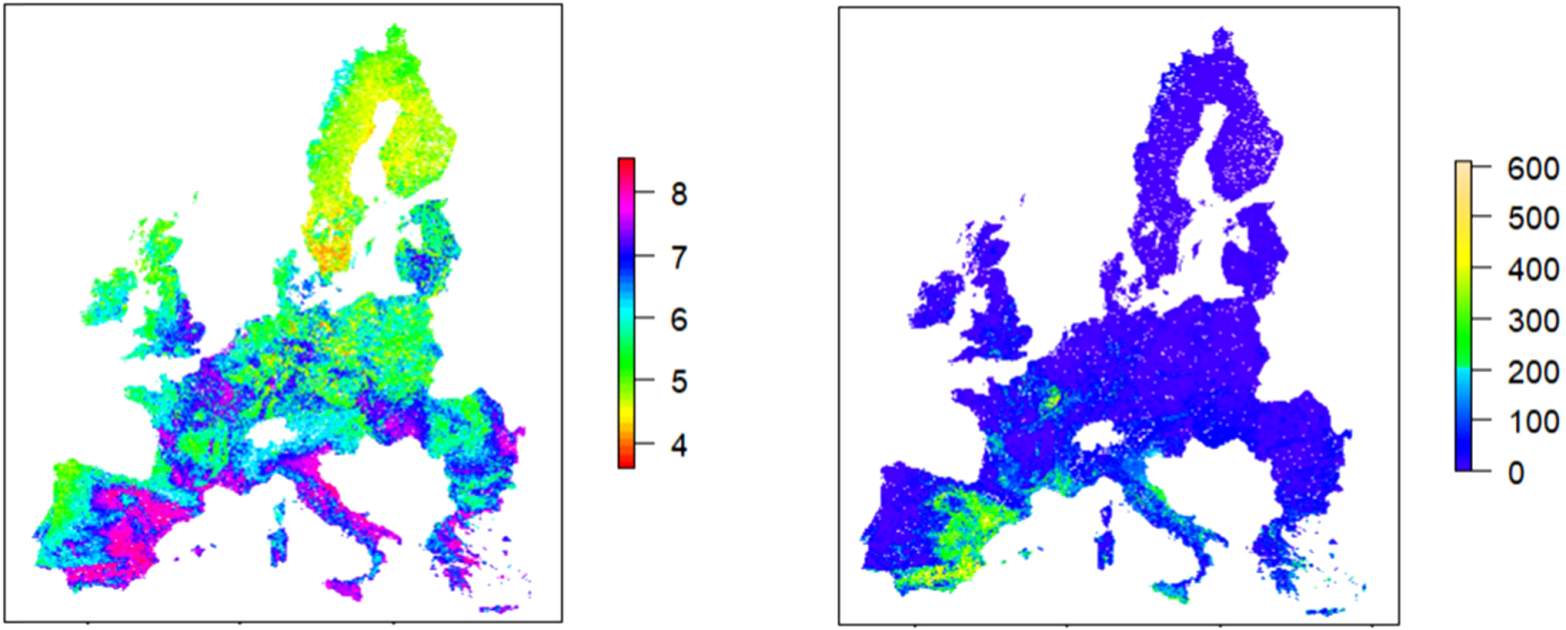
pH in H_2_O (left) and CaCO_3_ concentration (g · kg^-1^ of soil) (right) of Europe. Data have been provided by the European Soil Data Centre (ESDAC), and the maps have been made with R with the packages “terra” “tiff” and “raster”.

Across the Mediterranean and parts of Central Europe, soils are predominantly alkaline (pH 7.5–8.5), particularly in Spain, Italy, and Greece (**Figure 1**). These alkaline zones are typically rich in CaCO_3_ (**Figure 1**), which buffers soil pH and limits the availability of iron (Fe), thereby constraining crop yields. At such pH values, Fe solubility is minimal because of the low solubility of the existing Fe(III) oxides, oxyhydroxides, and hydroxides, so plants can hardly absorb it (Lucena, 2006). Iron deficiency in plants causes interveinal yellowing in young leaf areas (Álvarez-Fernández et al., 2011) because of the limitation of the chlorophyll synthesis and induce the stress of the plants by increasing the reactive oxygen species (ROS) production (Hajiboland, 2012), among other effects. Fe deficiency also promotes an increase in root diameter to enhance the capacity to reduce Fe(III) compounds (Pestana et al., 2012).

Plants have naturally evolved strategies to mitigate Fe deficiency. These strategies include Strategy I (dicotyledonous species, such as Cucumis sativum, and non-Gramineae monocotyledonous species) and Strategy II (Gramineae, such as rice), also known as the chelating strategy (Römheld and Marschner, 1986). In Strategy I, plants acidify the rhizosphere and release phenolic compounds or organic acids that facilitate the reduction of Fe(III) to Fe(II) (Kang et al., 2023). The enzyme Ferric Chelate reductase (FCR), encoded by *FRO2* (Robinson et al., 1999), reduces Fe in the root. The resulting Fe(II) is then transported across the epidermal plasma membrane primarily by the high-affinity Fe(II) transporter *IRT1* (Kobayashi and Nishizawa, 2012), with additional contributions (depending on species and conditions) from transporters encoded by distinct genes.

Under severe Fe deficiency conditions, the natural plant strategies are insufficient, and an external Fe supplementation, usually in the form of Fe chelates, is needed. Synthetic Fe chelates, such as Fe ethylendiaminetetraacetic acid (EDTA/Fe^3+^) or Fe ethylenediamine-N,N′-bis(2-hydroxyphenylacetic) acid (*o,o-*EDDHA/Fe^3+^), are commonly used due to their efficiency and versatility, being applicable via foliar spray or soil application (Lucena, 2006). However, in calcareous soils, their effectiveness can be limited by the chelate´s stability and its affinity for other cations, especially Ca^2+^. Chelates like *o,o-*EDDHA/Fe^3+^, with a higher stability constant but with low stability of the Ca chelate, perform better under calcareous conditions compared to EDTA/Fe^3+^, with a lower stability constant and with relatively high stability of its Ca chelate (**Table 1**).

**Table 1:** Synthetic chelating agents used in this paper, relevant Fe and Ca stability constants, and the pH range of stability of the Fe chelates in soils. Pyoverdine data are included for comparison.

Chelating agent	Abbreviation	Fe-chelates Log K°	Ca-Chelates Log K°	pH stability range
*Ethylendiaminetetraacetic acid*	EDTA	FeL 27.66 FeHL 29.17 FeOHL 19.84	CaL 12.44 CaHL 15.97	4 – 6.3 (Martell et al., 2004)
*Ethylenediamine-N,N′-bis(2-hydroxyphenylacetic)acid*	*o,o*-EDDHA	FeL 37.66 FeHL 39.67 FeOHL 25.80	CaL 9.00 CaHL 18.91 CaH_2_L 28.30	4 – 10 (Yunta et al., 2012)
*N,N′ - bis(2-hydroxybenzyl)ethylenediamine-N,N′ -diacetic acid*	HBED	FeL 42.25	CaL 11.00 CaHL 20.12 CaH_2_L 27.83	4 – 12 (Yunta et al., 2012)
*Pyoverdine*	Pvd	FeL 31.06 FeLH 40.23 FeLH_2_ 45.97 FeLOH 20.71		4 - 10.4 (Boukhalfa et al., 2006)
*desferrioxamine-B*	DFO-B	FeL 30.6	CaL 2.64	4 – 10.7 (Martell et al., 2004)

Despite their efficacy, synthetic Fe chelates pose environmental risks. They are poorly retained in soil, leading to potential leaching and contamination of water systems (Cesco et al., 2000). Furthermore, chelating agents like EDTA are highly persistent, altering soil chemistry and potentially mobilizing heavy metals, which can have harmful ecosystem effects (Tandy et al., 2004). To address both Fe deficiency and environmental concerns, the recent research has focused on biochelates, natural organic ligands capable of effectively binding Fe, such as plant-derived peptides or siderophores, among others (Di Palma and Mecozzi, 2007; Di Palma et al., 2011; Zuluaga et al., 2023).

The present work contributes to the existing knowledge in the field of siderophores as biochelators for use in alkaline soils. Siderophores, natural Fe chelators produced by microorganisms, have a high affinity for Fe (Neilands, 1984). However, studies evaluating the effectiveness of these compounds isolated from the bacteria under alkaline conditions are limited. Previous works have presented siderophores, such as azotochelin (produced by the nitrogen-fixing bacterium *Azotobacter vinelandii* (Bellenger et al., 2007; Ferreira et al., 2019) or a synthetic model of rhodotorulic acid (produced by *Rhodotorula pilimane*, N,N-dihydroxy-N,N’-diisopropylhexanediamide, DPH (Barclay et al., [[NoYear]])) as alternatives to synthetic chelates. They improved chlorophyll, natural Fe mobilization to the plant, and lengthened the effect in soybean plants cultivated in calcareous soil compared with synthetic chelates of *o,o-*EDDHA/Fe^3+^ and EDTA/Fe^3+^. Besides, López-Rayo et al (López-Rayo et al., 2019). studied the siderophore ethylenediaminedisuccinic acid ([*S,S´*]-EDDS), produced by the actinomycete *Amycolatopsis japonicum.* Although [*S,S*´]-EDDS/Fe³^+^ is less stable than synthetic chelates (Ahrland et al., 1990; Orama et al., 2002; Yunta et al., 2003), it effectively supplied Fe to plants in alkaline soils due to lower calcium (Ca²^+^) competition. Although a repeated dose is required to match the long-term effectiveness of *o,o*-EDDHA/Fe³^+^, its biodegradability means that degradation products still contribute to Fe availability, supporting its eco-friendly potential.

In the effort to identify a biochelate that exhibits reduced susceptibility to degradation in comparison to DPH/Fe^3+^ or AZO/Fe^3+^, and possesses a higher stability constant than [*S,S*´]-EDDS/Fe^3+^, pyoverdine (Pvd) has been under consideration as a potential siderophore that could fulfill these criteria. Pyoverdine is the most relevant siderophore produced by the bacterial genus *Pseudomonas* (Meyer and Abdallah, 1978). More than 60 Pvds have been chemically identified, having a three-component structure: (i) a chromophore (2,3-diamino-6,7-dihydroxyquinoline) responsible for its fluorescence; (ii) a peptide chain of variable length between 6 and 14 amino acids; and (iii) a side acyl chain attached to the chromophore (Cezard et al., 2015). Pyoverdine is considered a mixed siderophore, as it has catecholate and hydroxamate groups through which it binds to Fe (Soares, 2022). The high Fe chelating capacity of pyoverdine, expressed by its high stability constant (logK°_Pvd/Fe_ =40.23 (Boukhalfa et al., 2006)), highlights its potential use as a biochelate for plant fertilization.

In this line, a limited number of experiments have been reported showing the potential of this siderophore as a biofertilizer. In the study conducted by Gao et al (Gao et al., 2022). on apple rootstocks grown hydroponically under Fe-deficient conditions with pyoverdine supplementation, it was observed that pyoverdine addition mitigated chlorosis caused by Fe deficiency and enhanced Fe uptake. Similarly, Wang et al (Wang et al., 2024). investigated peanut-maize intercropping systems and found that the presence of *Pseudomonas* improved maize yield and Fe nutrition in calcareous soils. Their findings indicated that these benefits were associated with bacterial pyoverdine production, and they further observed that exogenous pyoverdine application in monocrops represents an effective and sustainable strategy to combat Fe deficiency. Finally, Lozano-González et al (Lozano-González et al., 2023). demonstrated the ability of pyoverdine to chelate Fe over a wide pH range, particularly under alkaline conditions.

Despite the beneficial effects of *Pseudomonas* or their secreted compounds on plants being well documented, only a limited number of studies have investigated the use of Pvd/Fe^3+^ as a biochelate. In fact, there is a lack of knowledge on the explanation of the mechanisms by which pyoverdine can enhance Fe acquisition in plants.

This study investigates the potential of the iron-complexed secretion from the *Pseudomonas monsensis* RMC4 strain, referred to as the Pvd/Fe³^+^ biochelate, to serve as an iron source for Strategy I plants under alkaline conditions. It is hypothesized that Pvd/Fe³^+^ not only provides a stable and efficient iron supply but also functions as a biostimulant, enhancing iron acquisition mechanisms and promoting plant health under stress. As preliminary steps before soil-based plant trials, the stability of Pvd/Fe³^+^ across different pH levels and in the presence of Ca²^+^ was assessed, alongside the application of the methodology proposed by Arcas et al (Arcas et al., 2024), which combines ferric chelate reductase (FCR) activity assays with ^57^Fe uptake analysis. Subsequently, the long-term effects of Pvd/Fe³^+^ application were evaluated in hydroponically grown plants under high pH conditions.

## 2 Materials and methods

### 2.1 Biochelate preparation

The *Pseudomonas* RMC4 bacteria were previously selected for its high pyoverdine production capacity (Lozano-González et al., 2023). Its genome has been uploaded to the NCBI database (https://www.ncbi.nlm.nih.gov/bioproject/PRJNA1028413/). Bacteria were grown in a 10% phosphorus minimal medium succinate (MMS) (Vindeirinho et al., 2021) to optimize siderophore production, following the procedure described by Lozano-González et al (Lozano-González et al., 2023). In brief, the bacteria-free solution (PsE), rich in pyoverdine and biostimulant metabolites, was used as the precursor for the Fe biochelate in this work.

The Pvd-based Fe biochelate (Pvd/Fe^3+^) was prepared by mixing the bacterial-free secretion (PsE) with a Fe salt solution. For that, the concentration of pyoverdine was first determined according to the law of Lambert Beer, already described at 400 nm (ϵ = 16,000 L · mol^-1^ · cm^-1^) (Meyer and Abdallah, 1978). Afterwards, a solution of Fe(NO_3_)_3_ · 9 H_2_O (Panreac, Barcelona, Spain) was added in a 1.02:1 pyoverdine:Fe (mol:mol) ratio. At the same time, pH was maintained in the range of 5.5-7.0 by adding a NaOH solution to ensure complete Fe chelation and prevent Fe precipitation, and was finally adjusted to pH 7.0. The solution was allowed to stand overnight, subsequently filtered through a 0.45 μm nylon membrane filter, and then made up to volume. The final concentration of Pvd/Fe^3+^ in the solution was determined in an Inductively Coupled Plasma Optical Emission Spectroscopy (ICP-OES, Thermo Fisher Scientific, Waltham, MA, USA) after acidification with HNO_3_, corresponding to 83 µM.

### 2.2 Preparation of Fe synthetic chelates

The synthetic chelates used for comparison, EDTA/Fe^3+^ and HBED/Fe^3+^, were prepared according to López-Rayo et al (López-Rayo et al., 2016). In brief, Na_2_EDTA·2H_2_O (Titriplex III, Merck) was directly dissolved in water, and HBED (kindly provided by ADOB PPC, Poznan, Poland (93.7%)) was dissolved in NaOH solution in a 1:4 ratio (ligand:base). Then Fe chelates were prepared by adding a solution of Fe(NO_3_)_3_ · 9 H_2_O, following the same procedure as with pyoverdine. The concentration of Fe in all the chelate solutions was also determined by ICP-OES after acidification with HNO_3_, corresponding to 1 mM.

### 2.3 Stability in solution versus pH and Ca^2+^ influence

The stability of the ferrated *Pseudomonas* RMC4 extract (Pvd/Fe^3+^), in comparison to EDTA/Fe^3+^ versus pH, was studied following the procedure described by Hernández-Apaolaza et al (Hernández-Apaolaza et al., 2006). with modifications. Ten milliliters of 83.0 µM Pvd/Fe^3+^or EDTA/Fe^3+^, 4 mL of 0.125 M CaCl_2_ · 2H_2_O (Merck), and 4 mL of the corresponding 0.0125 M buffer (pHs 5 and 6 with 2-(N-morpholino)ethane sulfonic acid [MES], pHs 7 and 8 with 4-(2-hydroxyethyl)-1-piperazineethanesulfonic acid [HEPES], pH 9 with 3-([1,1-dimethyl-2-hydroxyethyl]amino)-2-hydroxypropanesulfonic acid [AMPSO], and pH 10, 11, and 12 with 3-(cyclohexyl amino)propane-1-sulfonic acid [CAPS]) were added to a 50 mL volumetric flask. Then, 25 mL of distilled water was added, and the pH was adjusted using a pH meter (Thermo Scientific, Orion DUAL STARTM Meter) to 4.0, 5.0, 6.0, 7.0, 8.0, 9.0, 10.0, 11.0, and 12.0 with NaOH or HCl (concentrations of the base or acid between 0.001 and 1.0 M). After adjusting the volume, samples were transferred to 60 mL plastic vessels and were shaken at 56 rpm at 25˚C in darkness in an incubator (Boxcult J.P. SELECTA) for 3 days. Then, samples were filtered by a 0.45μm nylon membrane filter, pH was measured again, UV-Vis spectra of each sample were obtained (JASCO, UV-Vis/NIR Spectrophotometer V-650) between 200 and 700 nm, and the final Fe concentration in solution was determined using ICP-OES (Thermo Fisher Scientific, Waltham, MA, USA) after acidification. To study the Ca^2+^ interference in the Pvd/Fe^3+^ stability, an additional experiment was performed where the concentration of CaCl_2_ · 2H_2_O was modified. The entire protocol described above was carried out with pH adjusted to 8.0 (the value associated with the most pronounced behavior), and increasing concentrations of Ca^2+^ (0, 1.25, 2.50, 5.00, 7.50, or 10.0 mM) were added. Both experiments were conducted in triplicate.

### 2.4 Effect of the bacterial-free solution on seed germination and phytotoxicity

Fifteen cucumber (*Cucumis sativus* L. cv. Chinese Long) seeds were germinated in Petri dishes at increasing doses of the bacterial-free extract (PsE) corresponding to 0.0, 5.0, 25.0, or 40 µM pyoverdine. The seeds were allowed to germinate at 28˚C in an oven (IDL INDELAB) for three days. Afterwards, the number of sprouts was counted, and roots were scanned using an Epson Perfection V850Pro and analyzed by WhinRHIZO^®^ Pro. 2019 software (Regent Instruments, Canada). Relative germination (RG) (Equation 1), relative length (RL) (Equation 2), and Zucconi germination (GI) (Equation 3) indexes were calculated according to the following equations (Zucconi et al., 1981; Illera-Vives et al., 2011):

(1) $$Relative\;Germination\;\left(\text{RG}\right):\;\frac{number\;of\;germinated\;seeds\;in\;the\;treatment}{number\;of\;germinated\;seeds\;in\;the\;control\;treatment}\;\cdot 100$$

(2) $$Relative\;Length\;\left(\text{RL}\right):\;\frac{root\;length\;average\;in\;the\;treatment}{root\;length\;average\;in\;the\;control\;treatment}\;\cdot 100$$

(3) $$Zucconi\;Germination\;Index\;\left(\text{GI}\right):\;\frac{RG\;\cdot LR}{100}$$

### 2.5 Fe reduction and ^57^Fe absorption plant assays

The methodology already described for studying Fe chelates was applied with slight modifications (Arcas et al., 2024). Cucumber plants (*Cucumis sativus* L. cv. Chinese Long) were cultivated hydroponically inside a climate-controlled growth chamber (model CCKF 0/16985 Dycometal, Barcelona, Spain). The light regime consisted of 16/8 h day/night, with a temperature of 25/20˚C and relative humidity levels of 40/60%. The light intensity was 100 µmol m^-2^ s^-1^.

The seeds underwent a 5-day germination period in darkness at 25˚C, by being disposed in filter paper soaked with a 0.5 mM CaSO_4_ solution. Subsequently, twenty-four seedlings were transferred into plastic pots filled with 5 L of diluted nutrient solution (NS) at 1/5 strength. The NS had the following composition: macronutrients (Macro-NS, mM) 1.0 Ca(NO_3_)_2_, 0.9 KNO_3_, 0.3 MgSO_4_ · 7H_2_O, 0.1 KH_2_PO_4_, and micronutrients (Micro-NS, µM) 35.0 NaCl, 10.0 H_3_BO_3_, 0.05 Na_2_MoO_4_, 115.5 Na_2_-EDTA, 2.5 MnSO_4_, 1.0 CuSO_4_, 10 ZnSO_4_, 1.0 CoSO_4_, 1.0 NiCl_2_. During this period, 5.0 µM of Fe was added in the form of HBED/Fe^3+^, prepared as described in section 2.2. After 7 days, the diluted NS was replaced with full-strength NS, without any Fe addition to induce the Fe chlorosis of plants. Besides, to generate alkaline conditions, 0.1 g · L^-1^ of CaCO_3_ and 0.1 mM of HEPES (4-(2-hydroxyethyl)-1-piperazineethanesulfonic acid) buffered to pH 7.5 were also included in the NS composition. Plants grew for 16 (FCR assay) or 19 (^57^Fe absorption assay) days under these conditions.

#### 2.5.1 Ferric chelate reductase assay

The ability of Pvd/Fe^3+^ as a substrate for enzymatic reduction by the root-associated FCR was evaluated and compared to that of the chelate EDTA/Fe^3+^. For that, the roots of intact plants were washed with Macro-NS that also contained 37.5 µM Na_2_BPDS (disodium bathophenanthrolinedisulfonic acid). Then, individual replicates were prepared in a 250 mL beaker with 20 mL of Macro-NS, 0.33 mM MES (calculated concentration after dilution), buffered to pH 7.0, and 100 mL of a Pvd/Fe^3+^ or EDTA/Fe^3+^ solution of a concentration of 35.8 µM (calculated concentration after dilution), giving a total volume of 120 mL. The resulting Fe concentration was lower than that typically used for the FCR assays (100 µM (Arcas et al., 2024)) due to limitations related to the bacterial origin of the Fe substrate. The assay began with the simultaneous submersion of the root of one intact plant into the beaker and the addition of 0.72 mL of a 50 mM BPDS solution, resulting in a final concentration of 0.30 mM. Aliquots of 3 mL were collected at 0, 10, 20, and 60 min after the addition of BPDS. Seven independent plants (replicates) were prepared for each Fe treatment, and two blanks (without plants) for each. Fe(II)BPDS_3_ concentration in the solution was determined by calculations after absorbance determination (spectrophotometer UV-Visible Jasco V-650) at 535 nm (absorption maximum of Fe(II)-BPDS_3_ complex and 480 nm (absorption maximum of Pvd/Fe^3+^ chelate, not present in PsE) according to the equations described in Arcas et al (Arcas et al., 2024). methodology. Data were individually referred to the recorded fresh weight of the root.

#### 2.5.2 Absorption of isotopically stable labelled ^57^Fe

The capacity of cucumber plants to take up Fe from the ferrated *Pseudomonas* RMC4 extract (Pvd/Fe^3+^), and compared to EDTA/Fe^3+^, was evaluated using an isotopic labeling technique with the stable ^57^Fe isotope. Cucumber plants were germinated and pre-grown as described in section 2.5, but the Fe-deficiency period was maintained for 19 days. The assay was conducted with seven replicates per treatment and two blanks (without plants). The chelates were prepared as described in Section 2.2 but substituting Fe(NO)_3_ · 9 H_2_O with a solution of ^57^Fe (95.4%, Isoflex, Moscow, Russian Federation). Each beaker contained 120 mL of the solution described in the FCR assay but without BPDS, containing Pvd/^57^Fe^3+^ or EDTA/^57^Fe^3+^ at a final concentration of 35.8 µM (calculated after dilution). At time 0, the labelled Pvd/^57^Fe or EDTA/^57^Fe were applied. After 2h, plants were removed, roots, stem, and leaves separated, washed with a Tween solution (0.1% Tween 80, 1% HCl) and then twice with milli-Q water, weighed for fresh weight, and dried in an air-forced oven for 4 days at 60°C, to finally obtain the dry weight of the plants. Each plant-part sample was milled in a porcelain mortar and mineralized in a muffle oven at 480°C for 4 hours. Then, the ashes were dissolved in nitric acid (Suprapur, Merck) at a 1:1 ratio on a heating plate for 30 minutes. Samples were filtered (Whatman 1246) and made up to volume to obtain the final digested solution samples. Concentration of ^57^Fe and other stable Fe isotopes was quantified by ICP-MS (Inductively Coupled Plasma Mass Spectroscopy, Varian 820). Polyatomic interferences from Ca and Ar-based species were mitigated via the collision cell. The Fe from natural (Fe_nat_) and fertilizer (Fe_fert_) sources was determined after deconvolution by applying the procedure described by Rodríguez-Castrillón et al (Rodríguez-Castrillón et al., 2008). Finally, the Fe_Fert_ uptake rate of plants was calculated and expressed as the total µmol Fe · h^-1^ · g^−1^ root fresh weight, to be comparable to the FCR activity, which is also expressed in the same units.

### 2.6 Effect of the Fe biochelate Pvd/Fe^3+^ in Hydroponics at high pH and Fe levels

Cucumber plants (*Cucumis sativus* L. cv. Chinese Long) were germinated and hydroponically cultivated following the same methodology as that described in section 2.5. The Fe deficiency was induced for 5 days; after that, the plants were transferred to 500 mL plastic pots with full-strength NS, 0.10 g · L^-1^ CaCO_3_, and aerated. In this experiment, the effect of Pvd/Fe^3+^ application was compared to EDTA/Fe^3+^ (as in the previous experiments) and to HBED/Fe^3+^ at two Fe concentrations in the nutrient solution, 5 and 10 µM. Thus, the treatments were: 5 µM EDTA/Fe^3+^, 10 µM EDTA/Fe^3+^, 5 µM HBED/Fe^3+^, 10 µM HBED/Fe^3+^, 5 µM Pvd/Fe^3+^, 10 µM Pvd/Fe^3+^, and the control treatment without Fe addition (-Fe). These concentrations allow for the detection of physiological differences among treatments without inducing complete Fe sufficiency, which could obscure the comparative effectiveness of Fe fertilizers or chelates (Lucena and Chaney, 2006; Arcas et al., 2024). Five replicates of each treatment were carried out. The Macro-NS was renewed weekly and after 14 days plant sampling was carried out. During the experiment, the leaf chlorophyll index was assessed every two days for 14 days employing a digital chlorophyll meter, Soil Plant Analysis Development (SPAD) model 502 (Minolta, Co., Osaka, Japan), at each plant level. Level 2 (above the cotyledons) corresponded to growing leaves in the Fe-deficiency period (prior to treatments) that were sufficiently expanded on day 0 of the Fe treatments period. Level 3, where leaves initially grow at day 0 and are adequately developed 4 days after the application of the treatments. Finally, the development of level 4 leaves occurred approximately 10 days after treatment application. It could only be measured on the last day of the assay (day 14), except for control (-Fe) plants, which were not sufficiently developed due to the severe Fe chlorosis. On the plant sampling day, plants were washed and their fresh weight recorded as described in section 2.4. Five roots per treatment were also scanned and analyzed morphologically.

Reactive oxygen species (ROS) were assessed in five replicates of each treatment using 0.2 g (fresh weight, FW) of the youngest leaf, as described (Piterková et al., 2015) and (Hernández-Apaolaza et al., 2020). Leaves were chopped inside 2 mL of 50 mM HEPES at pH 7, then 50 µL of the extract was mixed with 150 µL of 50 mM HEPES and 4 µL of 5 µM H_2_DCFDA (diacetate of 2′,7′-diclorodihydrofluorescein) (Molecular Probes, Invitrogen, Carlsbad, CA, USA) and incubated for 30 min at 37 °C with orbital shaking at 100 rpm in the dark. Subsequently, the extract was centrifuged for 10 min at 1000 rpm, and the pellet was resuspended in 0.200 mL HEPES and incubated again for 10 min at 37 °C. DCF generated by the presence of ROS in the samples was monitored by recording the fluorescence intensity in an RF-600 spectrofluorometer (Shimadzu Corporation; Kyoto, Japan), setting the excitation wavelength at 488 nm and the emission wavelength at ≈525 nm. ROS levels were inferred from DCF fluorescence intensities.

The remaining plant material was dried and milled as described in 2.4. Finally, an ionomic analysis of leaves, stems, and roots was carried out on individual plant replicates by digestion of 0.5 g of dried milled material in a microwave (CEM Corporation MARS 240/50, Matthews, NC. USA) with 8 ml HNO_3_ 65% and 2 ml H_2_O_2_ 30% (Suprapur, Merck) with a program with a heating ramp up to 200 °C for 15 minutes, constant temperature for another 15 minutes and finally cooling, filtering and made up to volume. The nutrient concentrations were quantified in ICP-OES (Thermo Fisher Scientific, Waltham, MA, USA) after dilution.

### 2.7 Statistical analysis

Statistical analysis was performed using SPSS software for Windows (Version 24.0, SPSS Inc., Chicago, IL, USA). The Levene test for homogeneity of variances was first applied, followed by one-way analysis of variance (ANOVA). *Post-hoc* comparisons among treatments were then conducted using Duncan´s test, with a significance level set at p< 0.05.

## 3 Results

### 3.1 Stability of Pvd/Fe^3+^ in alkaline conditions: the role of pH and Ca^2+^

**Figure 2:**
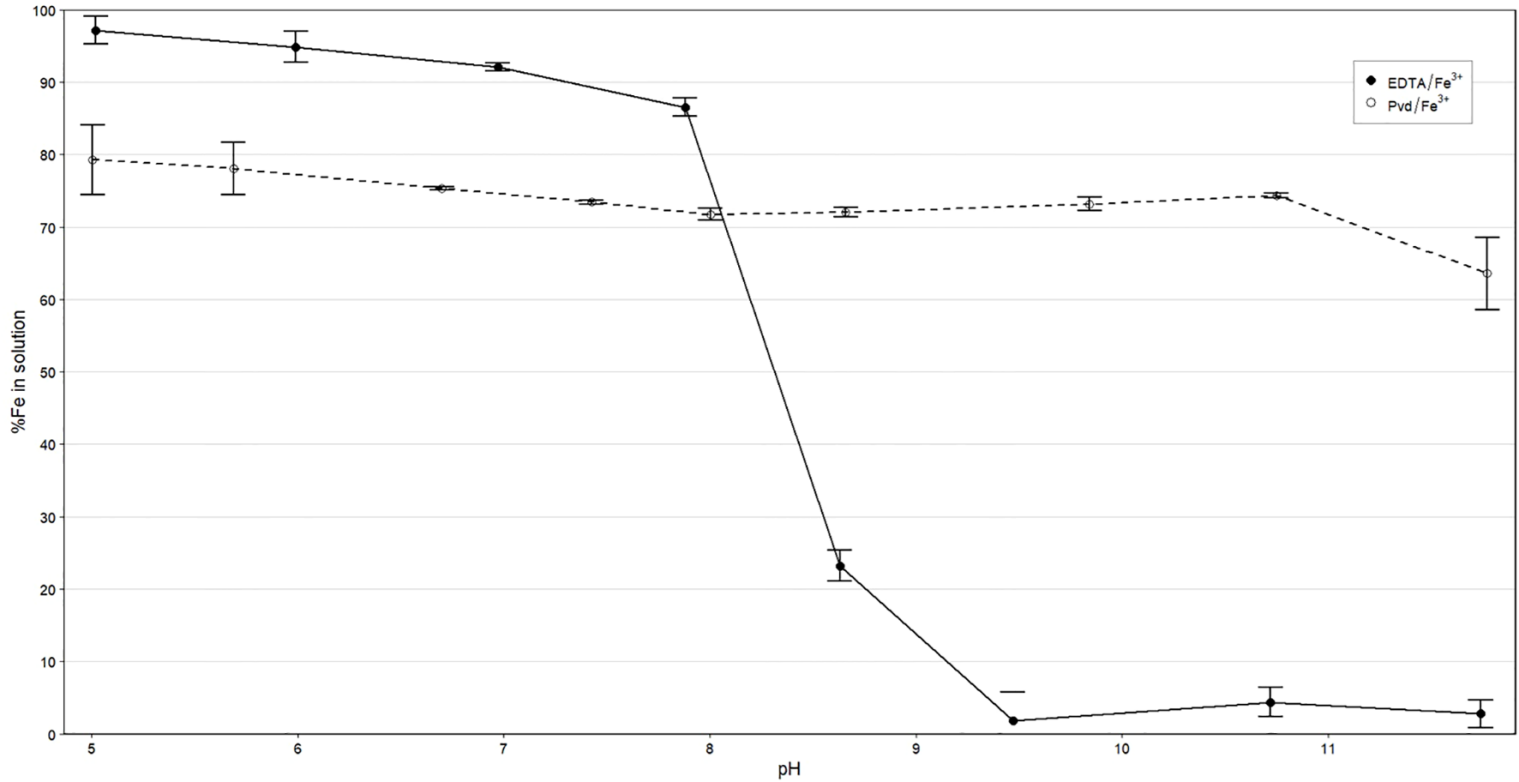
Percentage of soluble Fe recovered at different pH values in 10 mM Ca^2+^ solution. Data are the average (n=3) ± SE.

The stability of the Pvd/Fe^3+^ biochelate in the presence of 10 mM of Ca^2+^ was evaluated and compared to the synthetic EDTA/Fe^3+^ chelate over a pH range of 5 to 12 (**Figure 2**). The Pvd/Fe^3+^ biochelate maintained approximately 75% of the Fe in solution up to pH 11, where a slight decrease was observed up to pH 12. It can be observed that approximately 20% of the Fe in the biochelate was sensitive to rapid precipitation, likely due to other metabolites, such as PsE with a lower Fe affinity, which is why 100% of Fe was not obtained after the batch experiment at any pH. The stability of the EDTA/Fe^3+^ chelate is in agreement with previous studies, which show a notable decrease above pH 7, primarily due to the competition with Ca^2+^. This competence with Pvd/Fe^3+^ was further studied in an experiment conducted at different Ca^2+^ concentrations, with a fixed pH of 8.0 (**Table 2**). Generally, the effect of Ca^2+^ was significant at low pH levels. The Fe in solution was reduced by approximately 6% as the Ca^2+^ concentration increased up to 5 mM Ca^2+^, and by approximately 8% at higher concentrations. The results indicated that the Pvd/Fe^3+^ biochelate exhibited favorable stability in alkaline environments, maintaining Fe in solution.

**Table 2:** Concentration of soluble Fe (mg · L^-1^) of Pvd/Fe^3+^ biochelate at different Ca^2+^ concentrations (mM) and pH 8.0.

Concentration of Ca^2+^ (mM)	Concentration of soluble Fe (mg · L^-1^)
0.00	1.012 ± 0.035 **a**
1.25	0.954 ± 0.008 **b**
2.50	0.953 ± 0.002 **b**
5.00	0.928 ± 0.006 **c**
7.50	0.937 ± 0.003 **c**
10.0	0.935 ± 0.001 **c**

Data are the average (n=3) ± SE. Different letters in the same column indicate significant differences according to Duncan´s test (p< 0.05).

### 3.2 Phytotoxicity test of bacterial-free solution PsE

The possible phytotoxicity of the bacterial-free secretion of *Pseudomonas* RMC4 on cucumber seeds was evaluated at different pyoverdine concentrations using the Zucconi Germination Index (**Table 3**). Exposure of seeds to 5 µM or 25 µM PsE did not elicit any phytotoxic responses, while the highest dose of 40 µM reduced germination and root elongation.

**Table 3:** Germination parameters of cucumber seeds with different concentrations of *Pseudomonas* RMC4 extract (PsE) according to pyoverdine concentration.

Treatments	Relative Length	Relative Germination	Zucconi Germination Index
5 µM	107 **a**	85 ***ns***	91 **a**
25 µM	104 **a**	70	72 **a**
40 µM	60 **b**	77	46 **b**

Samples were collected after 3 days of germination at 28 °C. Data are the average (n = 5) ± SE. Different letters in the same column indicate significant differences according to Duncan´s test (p< 0.05). *ns* indicates no significant differences.

### 3.3 Plant response to Pvd/Fe^3+^

#### 3.3.1 Fe root reduction and ^57^Fe uptake by strategy I plants

The FCR assay yielded low FCR activity, resulting in values close to the limit of quantification when Pvd/Fe^3+^ was used as the Fe substrate (**Table 4**). The results obtained for the EDTA/Fe^3+^ used as a control were significantly higher than those for the Pvd/Fe^3+^, indicating a lower Fe reduction of the biochelate by the FCR enzyme. The values obtained for the EDTA/Fe^3+^ in our study conducted at pH 7 are in agreement with those reported in the literature, being higher at pH 6 (4.1 µmol Fe · h^-1^ · g^−1^ root fresh weight), but lower at pH 7.5 (0.33-0.7 µmol Fe · h^-1^ · g^−1^ root fresh weight) (Arcas et al., 2024). Despite this low FCR activity, the ^57^Fe absorption assay revealed that Fe provided by the Pvd/^57^Fe^3+^ (Fe_fert_) effectively entered the plant, even at a higher rate in the shoot, but achieved a lower total Fe in the plant than EDTA/^57^Fe^3+^ (**Table 4**). Comparing the resulting Fe_fert_ content in plant organs (**Table 5**), ^57^Fe from EDTA/^57^Fe^3+^ mainly accumulates in roots, while Pvd/^57^Fe^3+^ significantly improved Fe translocation to the shoot, underscoring the efficiency of the biochelate in promoting the Fe mobility within the plant.

**Table 4:** FCR activity and Fe_Fert_ uptake rate of plants, both expressed as µmol Fe · h^-1^ · g^−1^ root fresh weight, treated with Pvd/Fe^3+^ and EDTA/Fe^3+^ in the experiment of Fe Root reduction and ^57^Fe uptake.

Treatments	FCR assay	Absorption assays	
	FCR activity	Fe_Fert_ uptake rate (whole plant)	Fe_Fert_ uptake rate (shoot)
Pvd/Fe^3+^	0.5* ± 0.3	0.0120*** ± 0.0012	0.0022** ± 0.0004
EDTA/Fe^3+^	1.1 ± 0.2	0.0304 ± 0.0024	0.0012 ± 0.0003

Data are the average (n = 7) ± SE. *, ** and *** denote significant differences (p< 0.1, p< 0.05 and p>0.001 respectively) between treatments.

**Table 5:** Fe from Pvd/Fe^3+^ and EDTA/Fe^3+^ (Fe_fert_) in the different plant parts (nmol Fe ·plant^-1^) determined after two hours exposure to ^57^Fe treatments.

Absorption assays		
	Fe_fert_ nmol/plant	
Tissues	Pvd/Fe^3+^	EDTA/Fe^3+^
Leaves	2.0 ± 0.6 a	0.58 ± 0.16 b
Stem	1.7 ± 0.6 a	1.1 ± 0.3 b
Root	13 ± 2 b	45 ± 10 a

Data are the average (n = 7) ± SE. In the Fe_fert_ concentration results, significant differences (p< 0.05) between treatments for each plant part are indicated by letters as obtained by ANOVA.

#### 3.3.2 Pvd/Fe^3+^ effect in hydroponics at high pH and Fe levels

##### 3.3.2.1 SPAD index

SPAD dynamics in level 2 leaves showed no treatment differences from day 0-4. By day 8, all Fe-supplied treatments exceeded the -Fe control. HBED/Fe^3+^ (5 and 10 µM) consistently produced the highest SPAD values through day 14. The -Fe control declined steadily from 18.42 to 10.57 SPAD units (Δ = -7.85), indicating progressive chlorosis (see **Supplementary Table S1**, in **Supplementary Materials**), whereas chelated Fe maintained or increased SPAD. No chelated treatment matched the control´s loss; the most significant decline among them occurred with Pvd/Fe^3+^ 10 µM (Δ = -4.28), still substantially smaller than the control (see **Supplementary Table S1**). The comparative values of the SPAD indices obtained on day 14 are shown in **Table 6**.

**Table 6:** SPAD index of different leaf levels of cucumber plants after 14 days of the corresponding Fe treatment application.

Treatments	Level 2	Level 3	Level 4
[- Fe] Control	10.6 ± 3.9 **d**	9.1 ± 2.5 **c**	–
EDTA/Fe^3+^ 5 µM	15.5 ± 2.7 **cd**	15.1 ± 3.1 **ab**	15.8 ± 4.1 **b**
EDTA/Fe^3+^ 10 µM	19.0 ± 4.4 **abc**	20.3 ± 4.0 **a**	17.1 ± 2.3 **ab**
HBED/Fe^3+^ 5 µM	24.1 ± 0.8 **a**	18.3 ± 2.8 **a**	17.6 ± 3.9 **ab**
HBED/Fe^3+^ 10 µM	22.7 ± 4.8 **ab**	19.3 ± 1.9 **a**	22.0 ± 3.3 **a**
Pvd/Fe^3+^ 5 µM	18.1 ± 3.7 **bc**	12.2 ± 4.8 **bc**	7.9 ± 2.5 **c**
Pvd/Fe^3+^ 10 µM	14.8 ± 2.6 **cd**	9.3 ± 1.2 **c**	6.7 ± 3.6 **c**

The data represent the average (n = 5) ± SE. Different letters in the same column indicate significant differences according to Duncan´s test (*p* < 0.05).

**Figure 3:**
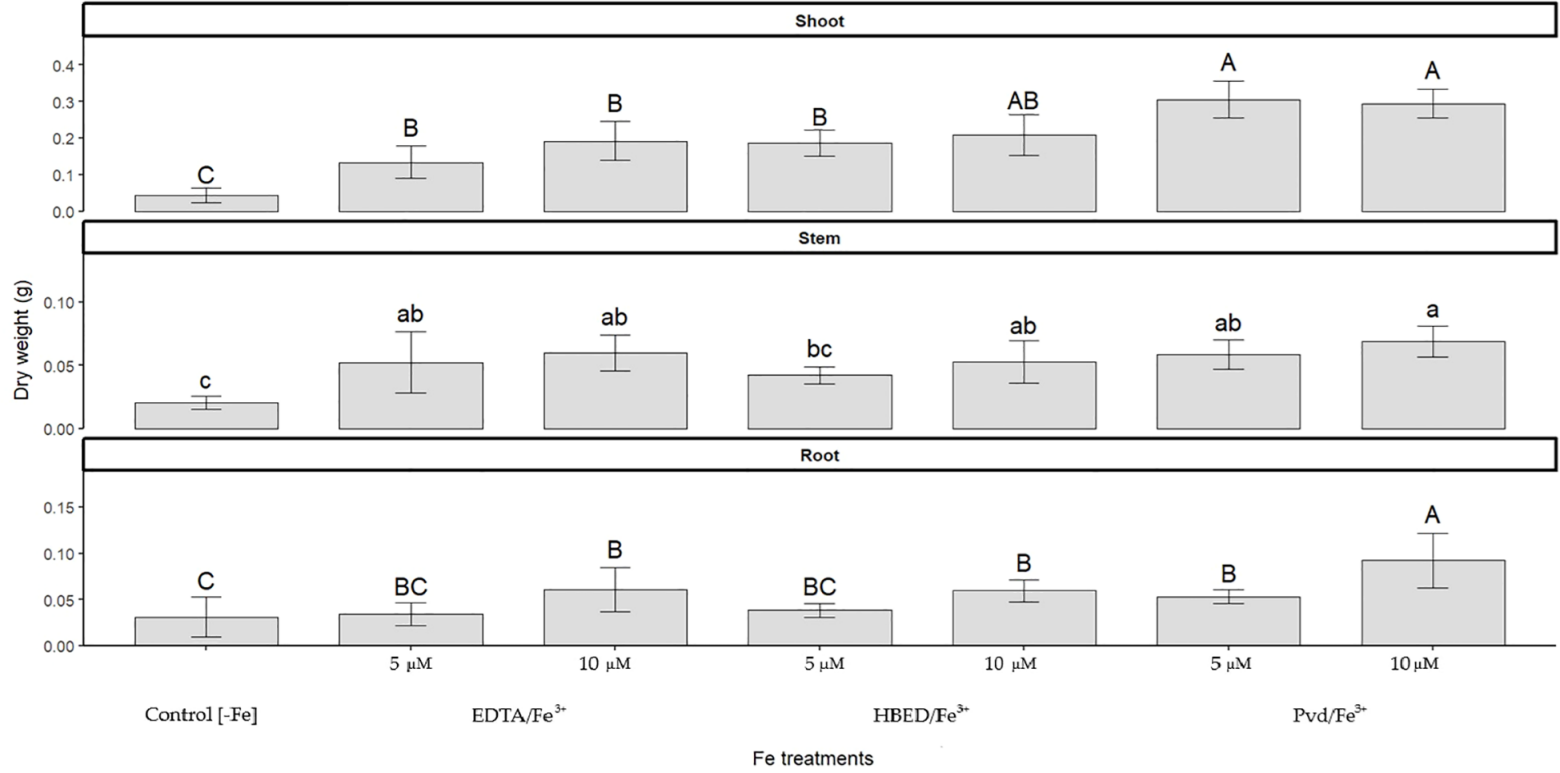
Dry weight of shoots, stems, and roots of cucumber plants grown under Fe-deficient conditions (Control [–Fe]) or supplied with Fe^3+^ as EDTA, HBED, or Pvd at 5 or 10 μM. Bars represent mean values (n=5) ± SE. Different letters within each panel indicate significant differences among treatments according to Duncan’s test (*p* < 0.05).

The SPAD index showed significant variation between Fe treatments and leaf levels. In the Fe-deficient control (-Fe), SPAD values were the lowest at all levels, with Level 4 leaves being absent due to restricted plant development (**Table 6**). This finding is indicative of the severe effect of Fe deficiency on leaf expansion and chlorophyll synthesis. The Pvd/Fe^3+^ treatments showed a decrease in SPAD values at the higher 10 µM dose in comparison to the 5 µM dose, particularly in level 3 and level 4 leaves. The decline in SPAD values likely reflects a dilution effect resulting from the substantial increase in biomass observed in the treatments (**Figure 3**). The iron supply by synthetic chelates improved the SPAD index, with variations depending on the Fe source and concentration. Among the treatments, HBED/Fe^3+^ 10 µM exhibited the highest SPAD values across all leaf levels, indicating superior Fe availability and uptake. EDTA/Fe^3+^ also increased SPAD values, but with slightly less efficiency than HBED/Fe^3+^. These results were consistent with those already reported in the literature for these Fe chelates.

##### 3.3.2.2 Root morphological analysis

The control –Fe plants exhibited the shortest root length (**Table 7**), thereby indicating the adverse effect of Fe deficiency on root elongation. The application of the Pvd/Fe^3+^ at 10 µM resulted in the longest roots, suggesting that this treatment effectively enhanced root elongation. The number of root tips, which indicates root branching and exploratory capacity, was significantly higher in all plants treated with Fe. Those treated with HBED/Fe^3+^ at 10 µM presented the highest values. The number of root forks, which denotes the points at which roots divide, was also significantly higher in the HBED/Fe^3+^ 10 µM.

**Table 7:** Morphological analysis (length (cm), number of tips, and number of forks) of the roots of cucumber plants 14 days after the application of the treatments.

Treatments	Root morphology		
	Length (cm)	Number of Tips	Number of Forks
[- Fe] Control	273 ± 6 **d**	384 ± 90 **c**	1642 ± 298 **d**
EDTA/Fe^3+^ 5 µM	361 ± 16 **bc**	2270 ± 455 **ab**	3849 ± 509 **c**
EDTA/Fe^3+^ 10 µM	347 ± 21 **bc**	1921 ± 364 **b**	5114 ± 635 **b**
HBED/Fe^3+^ 5 µM	327 ± 20 **c**	1722 ± 479 **b**	5320 ± 550 **ab**
HBED/Fe^3+^ 10 µM	342 ± 30 **bc**	3098 ± 660 **a**	6162 ± 227 **a**
Pvd/Fe^3+^ 5 µM	368 ± 19 **b**	1804 ± 324 **b**	5335 ± 398 **ab**
Pvd/Fe^3+^ 10 µM	421 ± 1 **a**	1790 ± 462 **b**	5697 ± 257 **ab**

Data are averages (n = 5) ± SE. Different letters in the same column indicate significant differences according to Duncan´s test (*p* < 0.05).

##### 3.3.2.3 Dry weight

At the end of the experiment, dry weight was measured (**Figure 3**). The plants treated with the biochelate Pvd/Fe^3+^, either at 5 or 10 µM, produced a significantly higher plant biomass than plants treated with conventional synthetic chelates. The effect of Fe deprivation caused the control plants to present a remarkably low dry mass. In detail, the dry mass of roots of the Pvd/Fe^3+^ 10 µM treatment showed higher values than the Pvd/Fe^3+^ 5 µM treatment, while the lowest values were found for – Fe, EDTA/Fe^3+^ 5 µM, and HBED/Fe^3+^ 5 µM treatments. The same trend could be observed in the stem, where the Pvd/Fe^3+^ 10 µM treatment showed the highest value. Finally, in the leaves, the Pvd/Fe^3+^ treatments (both Pvd/Fe^3+^ 5 µM and Pvd/Fe^3+^ 10 µM) showed higher values than the EDTA/Fe^3+^ treatments (EDTA/Fe^3+^ 5 µM and EDTA/Fe^3+^ 10 µM), HBED/Fe^3+^ 5 µM, and –Fe treatments, the control (-Fe) being the treatment that showed significantly lower values.

##### 3.3.2.4 Reactive oxygen species

**Figure 4:**
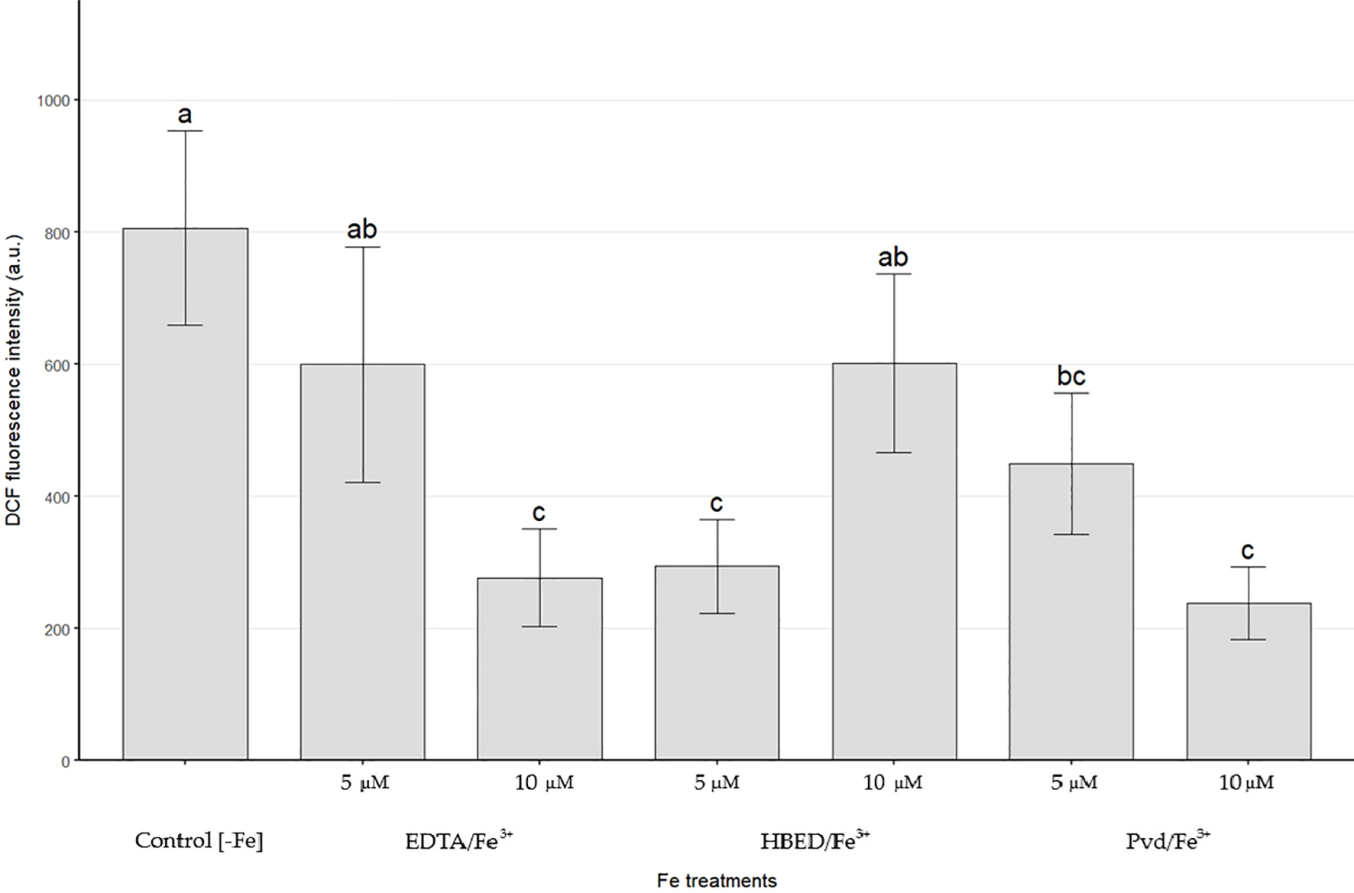
DCF fluorescence intensity in leaves of cucumber plants after 14 days of treatment with the different Fe sources. Relative ROS levels were inferred from the fluorescence intensity of 2',7'-dichlorofluorescein (DCF; excitation λ = 488 nm, emission λ ≈ 525 nm). Values are means (n=5) ± SE. Different letters indicate significant differences among treatments according to Duncan’s test (*p* < 0.05).

Leaf ROS was quantified by H_2_DCFDA oxidation to DCF. Fluorescence was used as a proxy for ROS/oxidative status (**Figure 4**). The –Fe control showed the highest signal, significantly exceeding EDTA/Fe³^+^ 10 µM, HBED/Fe³^+^ 5 µM, and PVD/Fe³^+^ (5 and 10 µM). It did not differ from EDTA/Fe³^+^ 5 µM or HBED/Fe³^+^ 10 µM. Among Fe-supplied treatments, the lowest ROS levels were observed for EDTA/Fe³^+^ 10 µM, HBED/Fe³^+^ 5 µM, and PVD/Fe³^+^ 10 µM.

##### 3.3.2.5 Ionomic analysis

The ionomic profiling of Fe-deficient plants treated with Pvd/Fe^3+^, compared to commercial Fe chelates (EDTA and HBED) and untreated controls, revealed distinct nutrient distribution patterns across leaves, stems, and roots (**Table 8**).

**Table 8:** Ionomic analysis expressed in µg · g^-1^ (Fe, Mn, and Zn) or mg · g^-1^ (P) of cucumber plants at day 14 of treatments.

Tissues	Nutrients	Ionomic analysis						
		[- Fe] Control	EDTA/Fe 5 µM	EDTA/Fe 10 µM	HBED/Fe 5 µM	HBED/Fe 10 µM	Pvd/Fe 5 µM	Pvd/Fe 10 µM
Leaves	Fe	43 ± 3 **c**	64 ± 7 **a**	59 ± 6 **ab**	67 ± 6 **a**	65 ± 7 **a**	42 ± 2 **c**	49 ± 8 **bc**
	Mn	40 ± 11 **c**	79 ± 2 **b**	81 ± 3 **b**	90 ± 8 **ab**	101 ± 6 **a**	24 ± 5 **d**	27 ± 6 **d**
	Zn	40 ± 2 **b**	59 ± 8 **a**	34 ± 3 **bc**	53 ± 6 **a**	54 ± 6 **a**	30 ± 7 **c**	26 ± 6 **c**
	P	3.21 ± 0.20 **d**	5.92 ± 0.62 **ab**	4.67 ± 0.60 **c**	6.70 ± 0.66 **a**	5.85 ± 0.34 **ab**	6.65 ± 0.85 **a**	5.57 ± 0.17 **b**
	Fe/Mn	1.08 ± 0.31 **ab**	0.79 ± 0.09 **b**	0.72 ± 0.08 **b**	0.74 ± 0.09 **b**	0.64 ± 0.08 **b**	1.74 ± 0.39 **a**	1.81 ± 0.51 **a**
	Fe/P	13.4 ± 1.3 **a**	10.6 ± 1.7 **ab**	12.5 ± 2.1 **ab**	10.0 ± 1.4 **b**	11.0 ± 1.4 **ab**	6.3 ± 0.9 **c**	8.7 ± 1.6 **c**
Stem	Fe	86 ± 15 **cd**	112 ± 24 **b**	98 ± 7 **bc**	170 ± 8 **a**	150 ± 7 **a**	62 ± 14 **de**	55 ± 4 **e**
	Mn	33 ± 3 **bc**	47 ± 8 **a**	45 ± 7 **ab**	38 ± 5 **ab**	42 ± 7 **ab**	19 ± 10 **d**	22 ± 11 **cd**
	Zn	76 ± 14 **b**	95 ± 12 **a**	84 ± 5 **ab**	93 ± 6 **a**	62 ± 10 **b**	67 ± 6 **b**	68 ± 8 **b**
	P	5.44 ± 0.75 **bc**	5.45 ± 0.67 **bc**	5.03 ± 0.52 **c**	5.95 ± 0.50 **ab**	6.20 ± 0.60 **ab**	6.44 ± 0.17 **a**	6.51 ± 0.30 **a**
Root	Fe	170 ± 6 **b**	183 ± 5 **a**	186 ± 2 **a**	197 ± 14 **a**	187 ± 8 **a**	197 ± 11 **a**	186 ± 7 **a**
	Mn	46 ± 6 **b**	62 ± 8 **a**	48 ± 5 **b**	47 ± 4 **b**	62 ± 5 **a**	46 ± 6 **b**	43 ± 9 **b**
	Zn	84 ± 8 **b**	105 ± 4 **a**	101 ± 5 **a**	102 ± 7 **a**	107 ± 7 **a**	96 ± 7 **ab**	104 ± 8 **a**
	P	2.98 ± 0.35 **bcd**	3.50 ± 0.50 **ab**	2.64 ± 0.35 **de**	2.18 ± 0.56 **e**	2.82 ± 0.23 **cd**	3.94 ± 0.38 **a**	3.41 ± 0.49 **abc**

Data are averages (n = 5) ± SE. Different letters in the same row indicate significant differences according to Duncan´s test (*p* < 0.05). Nutritional molar ratios were calculated from the micronutrients’ concentrations expressed in µmol · g^−1^ dry weight (Fe) or mmol · g^-1^ (P).

Leaf Fe concentrations under Pvd/Fe^3+^ were comparable to those observed with EDTA at 5 µM, and lower than EDTA at 10 µM and HBED at the two concentrations. Notably, Pvd/Fe^3+^ significantly reduced Mn accumulation in leaves, resulting in improved Fe/Mn ratios of 1.74 and 1.81 for 5 µM and 10 µM Fe, respectively. These values were substantially higher than those under EDTA and HBED (<1.0). This suggests a more favorable Fe/Mn nutritional balance, potentially mitigating Mn-induced Fe deficiency symptoms.

Leaf P concentrations were also elevated under Pvd/Fe^3+^, matching or surpassing those of commercial chelates, indicating enhanced nutrient balance. Although Fe/P ratios were lower, this reflects increased P availability. Zinc levels were similar to those under EDTA/Fe ^3+^ (10 µM) but lower than in other treatments.

In stems, Pvd/Fe^3+^ presented the lowest Fe concentrations, indicating a low accumulation in this plant tissue. Manganese levels were again reduced, consistent with leaf data. Pvd/Fe^3+^ also promoted the highest P accumulation in stems, suggesting improved nutrient translocation.

Root Fe concentrations under Pvd/Fe^3+^ treatments were similar to those of the synthetic chelates, without differences in the dose supplied. Manganese levels remained low, while Zn concentrations were consistently high in all the Fe-treated plants. Pvd/Fe^3+^ also led to the highest accumulation in roots.

To evaluate Fe supply efficiency, total Fe concentration across tissues was normalized to plant dry weight, corrected by subtracting values from Fe-deficient controls, and expressed as a percentage of Fe supply efficiency (**Table 9**). Pvd/Fe^3+^ (10 µM) demonstrated high Fe efficiency, comparable to HBED/Fe^3+^ (10 µM), and higher than EDTA/Fe^3+^. These results are consistent with the increased biomass observed under Pvd/Fe^3+^ (**Figure 3**), which probably led to a dilution effect on nutrient concentrations, thus explaining the moderate Fe levels reported in **Table 8**.

**Table 9:** Iron supply efficiency, expressed as a percentage of Fe supplied (calculated as total Fe content across tissues normalized to plant dry weight, and corrected by subtracting values from Fe-deficient controls).

Treatments	Efficiency (%)
EDTA/Fe^3+^ 5 µM	0.29 ± 0.01 **c**
EDTA/Fe^3+^ 10 µM	1.17 ± 0.01 **b**
HBED/Fe^3+^ 5 µM	1.87 ± 0.01 **a**
HBED/Fe^3+^ 10 µM	2.01 ± 0.01 **a**
Pvd/Fe^3+^ 5 µM	0.91 ± 0.02 **b**
Pvd/Fe^3+^ 10 µM	2.11 ± 0.01 **a**

Data are averages (n = 5) ± SE. Different letters in the same column indicate significant differences according to Duncan´s test (*p* < 0.05).

## 4 Discussion

Under alkaline conditions, Pvd/Fe³^+^ showed a largely pH-insensitive solubility profile: the fraction of Fe remaining in solution declined only from ~79% at pH 5.0 to ~64% at pH 11.8, whereas EDTA/Fe³^+^ remained high near neutrality but dropped sharply above pH 8 (**Figure 2**). These results indicate that Pvd/Fe³^+^ better preserves soluble (bioavailable) Fe across alkaline pH. For the correct interpretation of the results, the different chemical species of chelating agents formed, as a function of pH, will be discussed. The pyoverdines are a family of siderophores characterized by a common structure, comprising a chromophore, a peptide sequence, and an acyl side chain (Soares, 2022). The main difference in the interaction with Fe of the different pyoverdines lies in the peptide sequence, as it could interact with Fe via hydroxamate or α-hydroxycarboxylate groups.

In calcareous soils, the competition between Fe³^+^ and Ca²^+^ significantly impacts the stability of Fe chelates for most chelating ligands (Norvell, 1991; Álvarez-Fernández et al., 2002). The literature review agrees with the values obtained for EDTA/Fe^3+^ in **Figure 2**. From pH 7.8 onwards, the percentage of soluble Fe decreased drastically due to this Ca^2+^ competition. Regarding Pvd/Fe^3+^ biochelate, approximately 80% of soluble Fe was obtained until pH around 10.4. This percentage may correspond with the Fe chelated by Pyoverdine. The pyoverdine from *Pseudomonas putida* ATCC 33015 whose stability constants have been reported (Yunta et al., 2003) (**Table 1**), is expected to be similar to those contained in our bacterial-free extract from *Pseudomonas* RMC4 (PsE). The highly stable Fe chelate explains this behavior. To the best of our knowledge, there is no literature reporting the stability constant of pyoverdine with Ca^2+^. Moreover, studies conducted with desferoxamine-B (Martell et al., 2004), a trihydroxamic siderophore (data in **Table 1**), and rhizoferrin (Shenker et al., 1996; Willinger et al., 2015) indicated that these siderophores bind weakly to Ca^2+^, making Ca^2+^ competition with Fe^3+^ less significant than in the case of EDTA. This is also in good agreement with our results of the competition with Ca at pH 8 (**Table 2**). While around 80% of the complexing capacity of the PsE is due to the presence of pyoverdines able to strongly chelate Fe, around 20% of this complexing capacity should have a low Fe affinity. At the pH test conditions, this iron, weakly complexed, precipitates as observed in **Figure 2**.

Considering that the bacterial-free secretion of *Pseudomonas* (PsE) contains not only the siderophore pyoverdine but also other bioactive metabolites such as IAA or glutamic acid (Lozano-González et al., 2023), its application may exert both beneficial and adverse effects on seed germination and early seedling development. These compounds are known to influence plant physiological responses, including root elongation and hormonal signaling. In our study, the optimal concentration of PsE was identified between 5 and 25 µM, as determined by the Zucconi germination index (**Table 3**). However, at 40 µM PsE significantly inhibited germination and root elongation, suggesting a dose-dependent phytotoxic effect. It is important to note that such inhibitory responses at the seed level do not necessarily translate to similar impacts in mature plants, which possess more developed detoxification and regulatory mechanisms. Moreover, in practical applications, Fe would be supplied as a Pvd/Fe^3+^ complex, meaning that the concentration of free metabolites in PsE would be substantially lower, reducing the likelihood of phytotoxicity under standard cultivation conditions (Meliani et al., 2017; Waghunde and Sabalpara, 2021).

**Figure 5:**
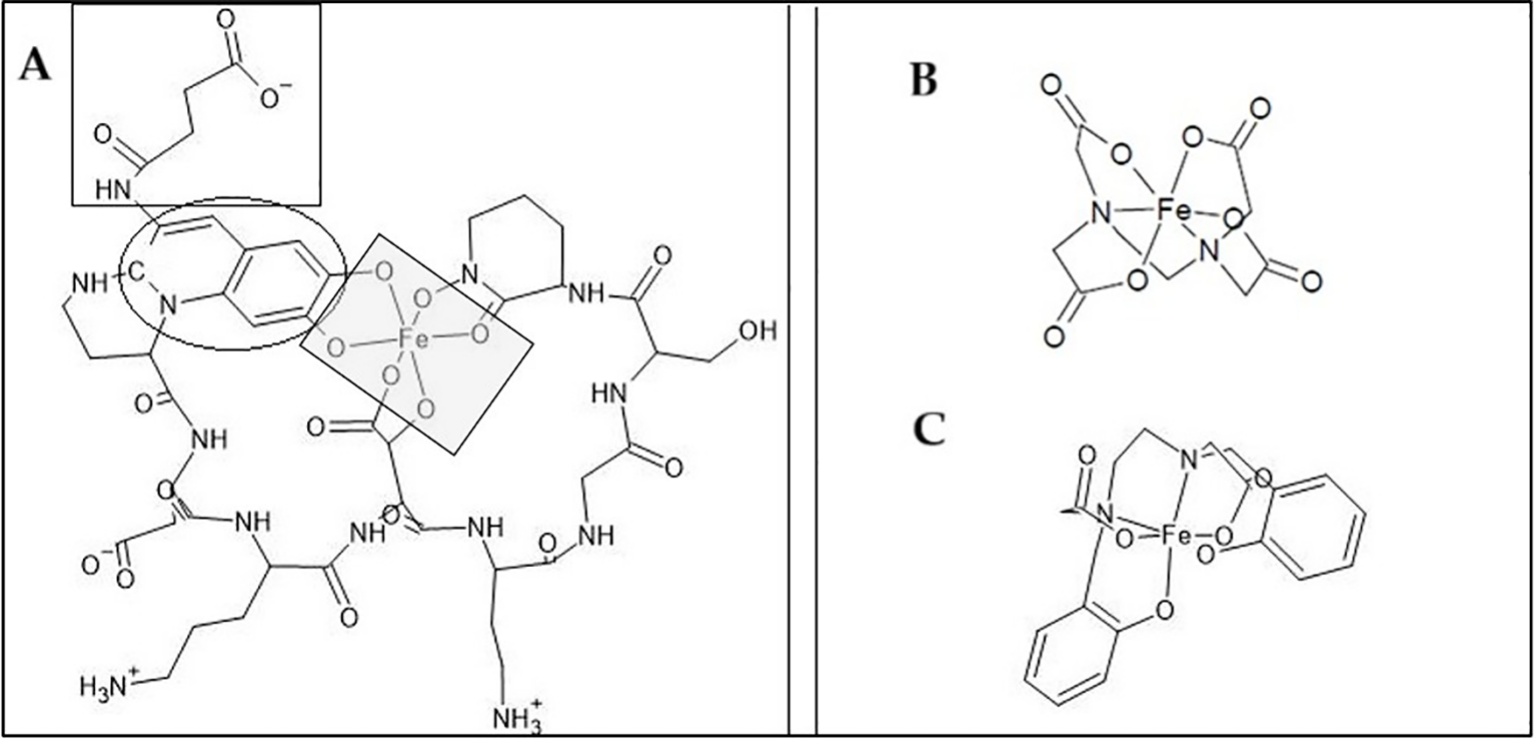
Structure of different Fe chelates. **(A)** Biochelate Pvd-Fe based on pyoverdine produced by *Pseudomonas putida* ATCC 33015, structure adapted from (Boukhalfa et al., 2006). The grey square represents Fe bonds, the black circle represents the chromophore group, and the black square represents the side acyl chain. **(B)** EDTA-Fe chelate. **(C)** HBED-Fe chelate. The structures were created using the software ACD/ChemSketch.

To evaluate whether Pvd/Fe³^+^ can serve as an Fe source for Strategy I plants, we contrasted FCR activity and Fe uptake (**Tables 4**, **5**) at pH 7. Pvd/Fe³^+^ yielded low FCR rates, indicating that Pvd-bound Fe is poorly reducible at the root surface or that Fe delivery may proceed via reduction-independent pathways; hence, FCR magnitude may not be a reliable proxy for Fe availability from this biochelate. Factors influencing the low FCR activity have already been discussed in the literature for other Fe chelates (Arcas et al., 2024). Since the optimal pH range for FCR is 6, and it decreases as pH increases, low enzyme activity values were expected at pH 7 (Ueno et al 2021). This diminution was also evident for EDTA/Fe^3+^, obtaining values in agreement with those already reported at pH higher than 6. Together with the pH, these observations support the view that chelate identity and speciation strongly condition the Strategy I response. FCR activity was monitored by the enzymatic reduction of Fe(III) to Fe(II), which BPDS subsequently sequesters to form the highly stable Fe(II)(BPDS)_3_ complex (log K° = 20.2 (Smith et al., 2021)), whose absorbance was measured spectrophotometrically. It could be hypothesized that the Pvd/Fe(II) complex, if sufficiently stable, might prevent the formation of Fe(II)(BPDS)_3_ and thus suppress FCR activity. However, the affinity constant of Pvd/Fe(II) (log K° = 9.0 (Cezard et al., 2015);) is markedly lower, so this possibility is dismissed. Although the chemical stability of Fe(III) chelates does not fully explain the variability in FCR activity (Arcas et al., 2024), it should be noted that highly stable chelates may promote the re-oxidation of Fe²^+^ to Fe³^+^ (Escudero et al., 2012). Furthermore, the FCR enzyme must access Fe for the reduction of Fe(III) to Fe(II). Open structures, such as *o,p*-EDDHA/Fe^3+^, lead to high values of FCR enzyme activity, whereas a more closed structure, such as HBED/Fe^3+^, results in lower values (García-Marco et al., 2006; Nadal et al., 2009). Considering these dimensional aspects, the plausible chemical structures of the pyoverdine contained in the studied extract, the Pvd/Fe^3+^ biochelate, and those of EDTA/Fe^3+^ and HBED/Fe^3+^ chelates are shown in **Figure 5**. The larger chemical structure with less accessible Fe is that of the Pvd/Fe^3+^. As illustrated in **Figure 5**, the large, sterically hindered structure of Pvd/Fe^3+^ likely prevents access to the FCR enzyme active site, explaining the low reduction activity. This suggests an alternative pathway for Fe uptake. Despite all this, chelates that have proven effective in correcting Fe chlorosis (HBED/Fe^3+^ or *o,o-*EDDHA/Fe^3+^), have not shown high FCR activity values (Lucena and Chaney, 2006; Nadal et al., 2009). Therefore, no correlation can be established between the values obtained in the FCR activity assay and the effectiveness of the chelate in providing Fe to the plant (Arcas et al., 2024). Furthermore, **Table 5** shows that EDTA/Fe^3+^ remains in the root, while Pvd/Fe^3+^ moves quickly to the shoots, suggesting either direct uptake or a different mobilization pathway. On the other hand, if the reduction is not occurring due to the stability of Pvd/Fe^3+^ or due to steric hindrance, the addition of reducing agents to the medium, along with Pvd/Fe^3+^, could be considered. This would assist the plant in the reduction process and facilitate the Fe^2+^ uptake. For instance, flavonoids could be beneficial, in addition to many other benefits for the plant (Samanta et al., 2011; Panche et al., 2016), although further research is needed to verify this hypothesis.

On the other hand, it has been reported that root exudation compounds such as coumarins or flavonoids, among others, can enhance Fe uptake when Fe is scarce (Clemens and Weber, 2016; Sisó-Terraza et al., 2016; Bosch et al., 2024), which may also contribute to explaining the FCR results.

The ^57^Fe absorption assay revealed that, while the EDTA/Fe^3+^ treatment led to a substantially higher concentration of ^57^Fe in the root, this was not the case for the stem and leaves (**Table 5**). This result suggests that Pvd/Fe^3+^ can supply Fe to the plant and is particularly effective in remobilizing this micronutrient to the aerial part. It is widely known that PGPBs influence the overexpression of genes involved in Fe-uptake responses, Fe transport, and storage (*FRO2, IRT1, FIT, MYB72*, among others) (Verbon et al., 2017; Oleńska et al., 2020). In the present study, the Pvd/Fe^3+^ biochelate contains not only pyoverdine but also the metabolites secreted by *Pseudomonas* RMC4, such as IAA and GABA, which have already been described (Lozano-González et al., 2023). These metabolites may have contributed to the overexpression of some of the genes mentioned above; therefore, Fe has undergone significantly higher translocation in the Pvd/Fe^3+^ treatment than in the EDTA/Fe^3+^ treatment. Another possibility is the uptake and translocation of the whole Pvd/Fe^3+^ biochelate. The addition of [^15^N]pyoverdine/Fe to the nutrient solution has been shown to result in their presence in the aerial part of the plant (Vansuyt et al., 2007; Shirley et al., 2011; Trapet et al., 2016). Despite the molecular mechanisms and transporters still being under study, this fact also supported the possible direct Pvd/Fe^3+^ uptake by the plant. Strategy I plants have been shown to be able to uptake and transport chelating agents at low concentrations (Orera et al., 2010), and pyoverdine is no exception (Vansuyt et al., 2007; Shirley et al., 2011; Trapet et al., 2016). It is plausible that a fraction of plant Fe was acquired as the Pvd–Fe³^+^ complex, which could account for the reduced chlorophyll content (SPAD) observed in newly emerged leaves (**Table 6**).

Regarding the plant response to Pvd/Fe^3+^ (section 3.3), a beneficial effect was observed, characterized by remarkable root development (**Table 7**) and a significant increase in biomass (**Figure 3**). The increased root development is probably due to the effect of the IAA present in the PsE, moreover, the mentioned results are in agreement with those described in the literature, where a significant increases in the biomass of tomato plants have been observed when applying 100 µM ferric Fe with pyoverdine (from *Pseudomonas fluorescens* ATCC 13525) than without pyoverdine (Nagata et al., 2013), or significant increase in *Arabidopsis thaliana* biomass has also been observed if pyoverdine, in its iron-depleted state (apo-pyoverdine), was introduced into the medium (from *Pseudomonas fluorescens* C7R12) (Trapet et al., 2016). On the other hand, lower oxidative stress, as indicated by lower ROS levels (**Figure 4**), has been observed. The metabolite GABA has likely mitigated oxidative stress by enhancing the plant´s antioxidant capacity. This effect could indicate the promotion of induced systemic plant resistance (IRS), an effect that has already been described in the genus *Pseudomonas* as well as when applying the microorganism or by applying apo-pyoverdine without microorganism (van Loon et al., 2008; Berendsen et al., 2015; Trapet et al., 2016). Regarding the effect of Pvd/Fe^3+^ on the Fe concentration in plants (**Table 8**), although it was lower than that of the synthetic chelates, in terms of efficiency (**Table 9**), the effect of the Pvd/Fe^3+^ was very positive, being similar to the Fe chelates. Despite higher total Fe accumulation in plants supplied with Pvd/Fe^3+^ 10 μM, SPAD chlorophyll index values remained low. This decoupling may reflect a growth-dilution effect and/or differences in Fe storage and speciation, including the possibility that a fraction of Fe persisted as the Pvd/Fe^3+^ complex rather than being mobilized into chloroplast-available pools. It is also remarkable that these plants exhibited a better nutrient balance, as indicated by the higher Fe/Mn ratio. No compelling results have been found in the literature in this regard. In the experiment performed by Trapet et al (Trapet et al., 2016), in *Arabidopsis thaliana*, it was observed that the Fe concentration in the roots 3 days after application of the treatments was lower in the apo-pyoverdine treatments than in their counterparts without pyoverdine, and no significant differences were observed in Fe content of the shoots. Furthermore, in the experiment conducted by Lurthy et al (Lurthy et al., 2020), using isolated pyoverdines from the rhizosphere of both Fe deficiency tolerant and susceptible varieties of pea (*Pisum sativum)*, it was concluded that the efficacy of these treatments in supplying Fe to a susceptible pea cultivar grown on calcareous soil depended on the pyoverdine source, with the cultivar-matched pyoverdine (isolated from the same cultivar) yielding superior results. They suggested that the effectiveness of the Pvd/Fe^3+^ biofertilizer depends on the producer bacteria, the crop to which it is applied, and the relationship between the bacteria and the crop. Although the present experiment differs from that described in terms of plant variety, mode of cultivation (soil vs hydroponics), and Fe concentration of the chelates, the similarities in the behavior of the Pvd/Fe^3+^ biofertilizer have been evidenced. In the present experiment, the Pvd/Fe^3+^ biofertilizer has been applied to cucumber plants (*Cucumis sativus* L. cv. Chinese Long). Although the effectiveness of the biofertilizer depends on the bacteria-crop relationship, in the case of *Pseudomonas* RMC4, it was initially isolated from pumpkin (*Curcubita*) (Lozano-González et al., 2023). Therefore, future assays should be conducted with the same plant variety from which the bacteria originate to verify whether the bacteria-crop relationship is decisive in the effectiveness of the biofertilizer.

This study demonstrated that a bacterial-free pyoverdine-based Fe biochelate (Pvd/Fe^3+^) can represent an effective alternative to synthetic Fe chelates in calcareous conditions. An effective Fe plant uptake has been determined; however, the evidence for FCR activity is unclear, suggesting that other mechanisms for Fe acquisition, which are still unknown for this biochelate, may also be involved. Additionally, the biochelate exhibited a biostimulation effect, increasing biomass, improving root morphology, and reducing ROS. In terms of Fe uptake efficiency, the Pvd/Fe^3+^ applied at a concentration of 10 µM showed a similar value to that of the synthetic chelate HBED/Fe^3+^; however, other parameters were not similarly improved at these Fe doses. Further studies to explore the mechanisms involved in uptake by the plant, as well as the bacteria-crop interaction, increasing the dose, and optimizing its efficiency with the addition of reductants to the medium, such as flavonoids, could help to better understand it. In addition, studies related to testing the biochelate in a real calcareous soil system are necessary to verify the potential of the Pvd/Fe^3+^.

## Data Availability

The datasets presented in this study can be found in online repositories. The names of the repository/repositories and accession number(s) can be found below: https://www.ncbi.nlm.nih.gov/, bioproject/PRJNA1028413/. Bacteria data are on that repository, but chemical data are in https://edatos.consorciomadrono.es/dataset.xhtml?persistentId=doi:10.21950/GFKHU3.
