# Supplementary material for: A pyoverdine-based iron biochelate from bacterial secretions as an effective fertilizer under alkaline conditions
Source: Front Plant Sci. 2025 Dec 15;16:1675837. doi: 10.3389/fpls.2025.1675837 (PMC12745479; doi:10.3389/fpls.2025.1675837)
Supplement: Supplementary file 1 [file DataSheet1.pdf]

Supplementary Material

A pyoverdine-based iron biochelate from bacterial secretions as an effective fertilizer under alkaline conditions

José María Lozano-González[1], Juan José Lucena[1], Sandra López-Rayo[1]*

[1] Department of Agricultural Chemistry and Food Science, Universidad Autónoma de Madrid, Av. Francisco Tomás y Valiente 7, 28049 Madrid, Spain.

[*] Correspondence: sandra.lopez@uam.es.

Table 1S. Time-course of the leaf SPAD index in level 2 of cucumber plants for each Fe treatment. Data are mean values (n = 5). Different letters in the same column indicate significant differences according to Duncan´s test ($p < 0.05$). *ns* indicates not significant differences.

Treatment	Day 0	Day 2	Day 4	Day 8	Day 11	Day 14
[- Fe] Control	18.4 *ns*	18.4 *ns*	17.2 *ns*	13.2 b	11.1 b	10.6 c
EDTA/Fe^{3+} 5µM	18.7	18.4	20.1	19.0 a	17.2 a	15.5 bc
EDTA/Fe^{3+} 10µM	18.5	19.3	19.1	19.7 a	19.2 a	19.0 ab
HBED/Fe^{3+} 5µM	18.5	22.9	20.5	21.5 a	21.7 a	24.0 a
HBED/Fe^{3+} 10µM	18.5	19.1	19.8	18.2 ab	20.7 a	22.7 a
PVD/Fe^{3+} 5µM	18.2	18.7	16.9	21.3 a	17.0 a	18.1 ab
PVD/Fe^{3+} 10µM	18.2	18.8	17.3	17.4 ab	16.8 a	13.9 bc
